# Synthesis and Gas Sensing Properties of Single La-Doped SnO_2_ Nanobelts

**DOI:** 10.3390/s150614230

**Published:** 2015-06-16

**Authors:** Yuemei Wu, Heng Zhang, Yingkai Liu, Weiwu Chen, Jiang Ma, Shuanghui Li, Zhaojun Qin

**Affiliations:** Institute of Physics and Electronic Information Technology, Yunnan Normal University, Kunming 650500, China; E-Mails: wuyuemei893@163.com (Y.W.); zhangheng214168@163.com (H.Z.); cww11022@126.com (W.C.); mayului@126.com (J.M.); lishuanghui808@163.com (S.L.); qinzhaojun031@163.com (Z.Q.)

**Keywords:** La-doped SnO_2_ nanobelts, single nanobelt, gas sensor, ethanediol

## Abstract

Single crystal SnO_2_ nanobelts (SnO_2_ NBs) and La-SnO_2_ nanobelts (La-SnO_2_ NBs) were synthesized by thermal evaporation. Both a single SnO_2_ NB sensor and a single La-SnO_2_ NB sensor were developed and their sensing properties were investigated. It is found that the single La-SnO_2_ NB sensor had a high sensitivity of 8.76 to ethanediol at a concentration of 100 ppm at 230 °C, which is the highest sensitivity of a single SnO_2_ NB to ethanediol among three kinds of volatile organic (VOC) liquids studied, including ethanediol, ethanol, and acetone. The La-SnO_2_ NBs sensor also exhibits a high sensitivity, good selectivity and long-term stability with prompt response time to ethanediol. The mechanism behind the enhanced sensing performance of La-doped SnO_2_ nanobelts is discussed.

## 1. Introduction

In recent years, metal oxide semiconductors with one-dimensional (1D) nanostructures such as nanowires, nanobelts, and nanotubes have been demonstrated to be promising candidates for ultrasensitive sensors because of their single crystal nanostructure, high surface-to-volume ratios, special physical and chemical properties [1,2]. Among them, SnO_2_, a well-known n-type semiconductor with band gap E_g_ = 3.6 eV at 300 K, is considered as the most promising functional material due to its highly sensing properties [3,4]. Hoa *et al.* reported that monolayer graphene (GP)/SnO_2_ nanowire (NW) Schottky junction devices could detect NO_2_ at a ppb level with detection limit of about 0.024 ppb [5]. However, tin oxide has problems related to its poor selectivity towards various gases. In general, suitable catalysts, noble metals, and transition metals are inserted into SnO_2_ sensors to improve their selectivity and sensing response [6,7,8]. These metals’ catalytic activities, coupled with the semiconductor properties of the materials used, have resulted in their applications for detection of organic, inorganic vapors, and other toxic, inflammable or hazardous gases [9,10]. For instance, Kim *et al.* reported that the doping of Ru into hollow spheres leads to the selective and sensitive detection of trimethylamine with negligible cross-responses to toluene, benzene, NH_3_, CO, H_2_, and C_3_H_8_ [11]. Hybrid SnO_2_/carbon nanotubes present a high sensitivity to O_3_ and NH_3_ at room temperature [12]. The sensor array composed of platinum-, copper-, indium-, and nickel-doped tin oxide nanowires has the capability of classifying organic vapors (chloroform, ethyl acetate, isopropanol, and methanol) [13].

The merit of SnO_2_ doped by noble metals is that its gas-sensing sensitivity and selectivity can be controlled by the addition of various noble metal catalysts. Our research group has found that a single Pd-doped SnO_2_ nanoribbon has high sensing properties to ethanol with high selectivity at 230 °C [14]. Recently, we have been studying the influence of rare earth elements on the sensing properties of SnO_2_ NBs and found that La-doped SnO_2_ NBs have a better response and selectivity to ethanediol. Therefore, we systemically investigated the sensing properties of a single La-SnO_2_ NB sensor to volatile organic (VOC) liquids and reported our interesting results in this paper.

## 2. Experimental Section

### 2.1. Synthesis of SnO_2_ NBs and La-SnO_2_ NBs

Monocrystal SnO_2_ and La-SnO_2_ NBs were obtained by the thermal evaporation method [15,16]. For synthesis of La-SnO_2_ NBs, a mixture of pure SnO_2_ powder (>99.99 wt%) and La_2_(C_2_O_4_)_3_·10H_2_O powder premixed in the weight ratio of 20:1 was put into a ceramic boat. The ceramic boat was placed in the central position of a horizontal alundum tube, which was put into a high temperature furnace. A silicon substrate coated with about 10 nm Au film was placed into the tube; the distance of silicon substrate and ceramic boat was about 10 cm. After cleaning the tube several times with nitrogen gas, the tube was evacuated by a mechanical pump to a pressure of 1 to 5 Pa. The SnO_2_ and La_2_(C_2_O_4_)_3_·10H_2_O powder precursors were evaporated at 1350 °C for 2 h and deposited on the Si substrate with Ar carrier gas (30 sccm, the pressure inside the tube is 125 Torr). After the furnace was naturally cooled to room temperature, white wool-like products were obtained, which were La-SnO_2_ NBs. In order to compare the sensing properties of La-SnO_2_ NBs and pure SnO_2_ NBs, we also prepared pure SnO_2_ NBs by a similar method.

### 2.2. The Characterization and Preparation of a Single Nanobelt Device

The nanobelts were characterized by scanning electron microscopy (SEM) and energy-dispersive X-ray diffraction (EDX). The microstructures of the nanobelts were analyzed by transmission electron microscopy (TEM) and high-resolution electron microscopy (HRTEM).

SnO_2_ NBs and La-SnO_2_ NBs were picked out and then dispersed into ethanol by tweezers. A few of the resulting suspensions were dropped onto a silicon substrate with a 500-nm-thick SiO_2_ layer. The suspensions were dried naturally, leading to nanobelts closely stuck to the substrate. A mask plate was placed on the top of this substrate to prepare the electrodes. Patterned Ti (20 nm) and Au (150 nm) electrodes were successively deposited on the nanobelts under high vacuum by dual-ion beam sputtering (LDJ-2a-F100-100 series) with Ar carrier gas (10 mA/cm^2^, 2.7 × 10^−2^ Pa). 

### 2.3. The Measure of Gas Sensitivity

**Figure 1:**
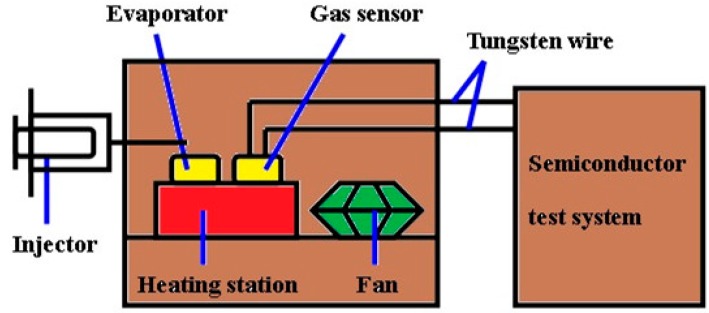
Schematic diagram of the test system.

The gas sensor measurements were performed with an equipment setup designed by our laboratory, as shown in Figure 1. The process was conducted in a hermetic stainless steel box (20 L). The device was put on a heating station, on which its temperature can be accurately controlled. The sensing properties of the device were measured by a Keithley 4200 semiconductor test system. The testing bias voltage was 1 V and the testing interval was 200 s. The target liquid can be injected into an evaporator to rapidly evaporate the VOC liquid and a fan is used to produce a homogeneous atmosphere in the chamber [17]. 

## 3. Results and Discussion

### 3.1. Structural Characterization and Microstructure Analysis 

**Figure 2:**
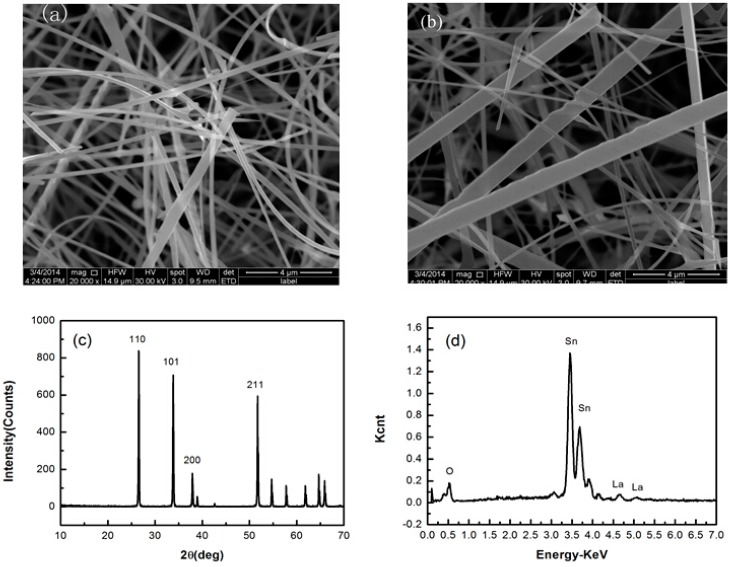
SEM images of La-SnO_2_ NBs (**a**); SEM images of SnO_2_ NBs (**b**); XRD and EDX patterns of La-SnO_2_ NBs (**c**) and (**d**), respectively.

The morphology of the as-synthesized materials as observed by scanning electron microscopy is displayed in Figure 2. The product of La-SnO_2_ and pure SnO_2_ consists of a large quantity of belt-like structures and wire-like ones, in which the nanowires have a different diameter, as shown in Figure 2a,b. Most nanobelts have this uniform thickness and width. Figure 2a shows that their thickness is less than 100 nm, the width is from 250 nm to 1 μm, and the length is about 50 μm. It is also seen that the obtained La-SnO_2_ NBs not only have good shape but also a smooth surface, which is suitable for preparing gas sensors.

The XRD pattern of the La-SnO_2_ NBs is presented in Figure 2c. The diffraction peaks can be indexed as the tetragonal structure SnO_2_ with lattice parameters a = b = 0.4736 nm, c = 0.3188 nm (JCPDS file No. 02-1340). No other materials such as La or related La oxides are detected. The reason is that the content of La doping is too low.

In order to know whether La^3+^ ions were doped into SnO_2_ NBs or not, the energy-dispersive X-ray diffraction (EDX) pattern of a single La-SnO_2_ NB was recorded, as shown in Figure 2d. It is seen that the doping content of SnO_2_ NBs is only 0.51 wt%.

**Figure 3:**
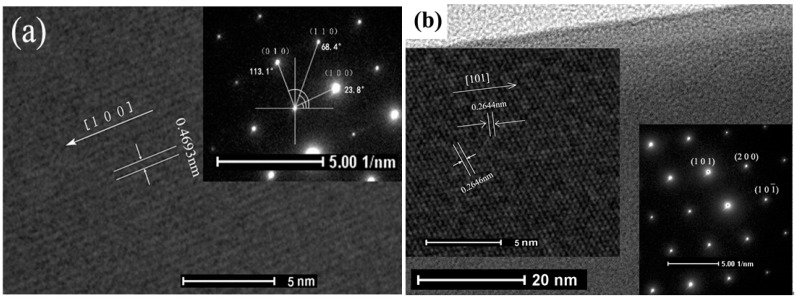
The HRTEM and SEAD images of pure SnO_2_ NBs (**a**) and La-SnO_2_ NBs (**b**).

For further insight into the microstructures of SnO_2_ NBs and La-SnO_2_ ones, HRTEM images and selected area electron diffraction (SAED) patterns of a single SnO_2_ NB and La-SnO_2_ one were obtained and are shown in Figure 3a,b, respectively. The lattice spacing between the adjacent planes is 0.4693 nm, corresponding within the measurement error to the d(100) interplanar spacing. The left inset of Figure 3b shows that the interplanar spacings between the adjacent planes are 0.2646 nm and 0.2644 nm, respectively, which correspond to (101) and the (10$\bar{1}$) crystal planes. Their selected-area electron diffraction (SAED) patterns in the right insets of Figure 3a,b were indexed to a tetragonal structure with a = b = 0.4736 nm, c = 0.3188 nm. Comparison of the HRTEM and SAED results reveals that the growth directions of SnO_2_ NB and La-SnO_2_ NB are along [100] and [101] from the edge of a nanobelt, respectively. Besides the growth direction, we have not found any influences of La^3+^ ions on the obtained sample’s microstructure.

**Figure 4:**
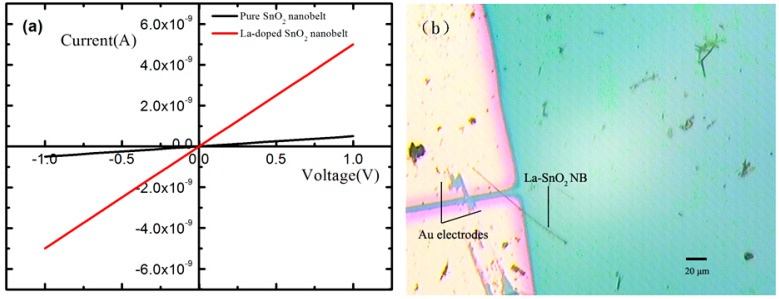
(**a**) The I–V curves of pure SnO_2_ NB and La-SnO_2_ NB devices; (**b**) The optical microscope image of a prepared La-SnO_2_ NB device.

Figure 4 present typical I–V curves when the devices were in air at room temperature. The approximately linear shape of the curves reveals good Ohmic contacts of SnO_2_ NB/La-SnO_2_ NB with the electrodes. The slope of pure SnO_2_ NB is less than that of the La-SnO_2_ NB. The resistance of La-SnO_2_ NB is about 2.05 × 10^8^ Ω and that of pure SnO_2_ NB is about 2.08 × 10^9^ Ω, indicating that the resistance of SnO_2_ is greatly reduced after doping. Figure 4b presents a typical optical microscope image of the obtained La-SnO_2_ NB device, which is composed of an individual nanobelt and Au electrodes.

### 3.2. Sensing Properties of Single La-SnO_2_ NB Device

The gas sensor sensitivity S is defined as follows:

S = R_a_/R_g_

where R_a_ is the sensor resistance in air (base resistance) and R_g_ is the resistance in a mixture of target gas and air. In addition to gas sensor sensitivity, the sensing properties of metal oxide semiconductors also can be characterized by the other parameters such as response time (T_res_) and recovery time (T_rec_). The response time and recovery time are defined as the time taken by the sensor to achieve 90% of the total resistance change in the case of adsorption and desorption, respectively [18]. 

#### 3.2.1. Working Temperature

**Figure 5:**
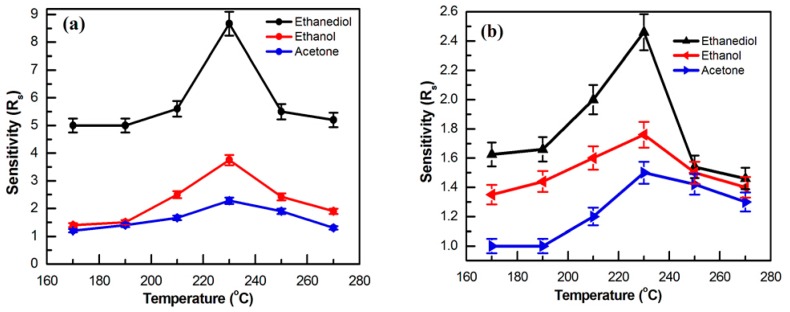
The gas sensitivity of (**a**) the La-SnO_2_ NB and (**b**) the SnO_2_ NB to 100 ppm gas from 170 °C to 270 °C.

The sensitivity of the sensors based on La-SnO_2_ NB and its undoped counterpart when exposed to 100 ppm of ethanediol, ethanol, and acetone gases has been tested as a function of operating temperatures in the range of 170 °C to 270 °C, as shown in Figure 5. The results indicate that the working temperatures greatly affect the sensitivity of two devices. As evidenced from Figure 5a, the optimum working temperature of the La-SnO_2_ NB sensor towards a level of 100 ppm ethanediol, ethanol, and acetone gases is 230 °C, at which the corresponding S values are 8.76, 3.75 and 2.28, respectively. The optimum working temperature of the SnO_2_ NB sensor (in Figure 5b) is 230 °C, where its S values when exposed to 100 ppm of ethanediol, ethanol and acetone gases are reduced to 2.46, 1.76, and 1.50, respectively. The results reveal that response of the La-SnO_2_ NB sensor to ethanediol gas is higher than that of the undoped counterpart (SnO_2_ NB). 

#### 3.2.2. High Response

**Figure 6:**
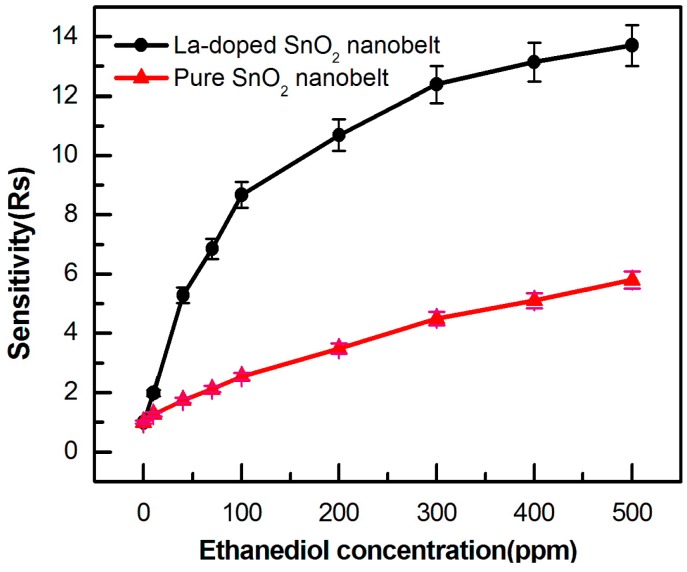
The gas sensitivity of two devices to ethanediol from 5 ppm to 500 ppm at 230 °C.

To explore their sensitivity, the responses of La-SnO_2_ NB sensor and SnO_2_ NB sensors is further investigated as a function of ethanediol gas concentration at 230 °C. The results indicate that the sensitivity increases with an increasing ethanediol concentration from 5 ppm to 500 ppm, as shown in Figure 6. This shows that La-SnO_2_ NB sensor has a remarkably higher response than the SnO_2_ NB sensor. It is noted that its response is first increased drastically in the range of 5–100 ppm, then moderately in the 100–300 ppm range, and finally slowly in the 300–500 ppm one. Furthermore, the minimum detection limit of the La-SnO_2_ sensor is around 5 ppm. As reported in the literatures, a CuO nanocubes sensor exhibited a high-sensitivity of ~132.84 ± 0.02 mA·cm^−2^·(mol/L)^−1^ and detection limit of ~5 × 10^−9^ mol/L toward 4-nitrophenol [19]. A sensor based on α-Fe_2_O_3_ nanoparticles (SnS_2_ nanoflakes) possessed a high sensitivity of ~367.6 (~505.827 ± 0.02) mA·cm^−2^·(mol/L)^−1^ with detection limit of ~1.56 × 10^−3^ (~15 × 10^−6^) mol/L toward 4-nitrophenol (nitroaniline) [20], and a fabricated hydroquinone chemical sensor of Ce-doped ZnO nanorods exhibited a sensitivity of ~10.218 ± 0.01 mA·cm^−2^·mM^−1^ with ~10 nM detection limit [21]. Therefore, sensors based on nanostructured materials have a very high sensitivity and lower detection limit toward VOC gases and hazardous chemical pollutants.

**Figure 7:**
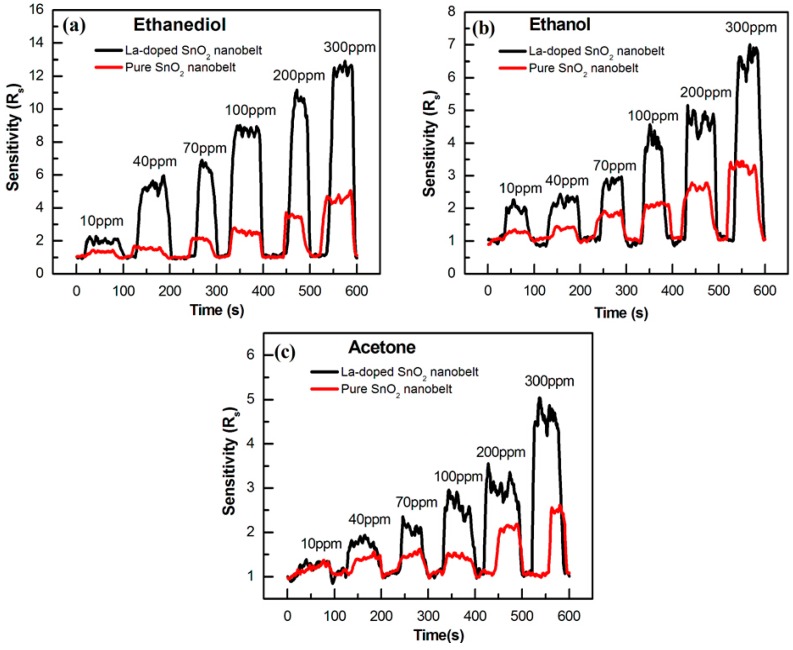
The gas sensitivity of two devices to (**a**) ethanediol; (**b**) ethanol and (**c**) acetone from 10 ppm to 300 ppm at 230 °C.

More details of the dynamic response of these sensors are provided upon repeated ethanediol, ethanol, and acetone gas exposure/removal cycles, as displayed in Figure 7. Six cycles are successively recorded, corresponding to 10, 40, 70, 100, 200 and 300 ppm of ethanediol, ethanol, and acetone gases, respectively. For all tested cycles, the resistance returns completely to its original value once the gases are pumped out. It can be seen that La-SnO_2_ NB has a high sensitivity and selectivity to ethanediol at all concentrations of the three gases. Combined with the different temperature response, it can be concluded that La-doped SnO_2_ NB can greatly enhance gas sensitivity and selectivity towards ethanediol.

#### 3.2.3. Response Time and Recovery Time

The response (recovery) time can provide the dynamic response of the sensors upon adsorption and desorption, respectively, which is an important parameter for electronic sensors. Response time and recovery time are difficult to measure accurately based on the fact that the air inflation and exhaust needs a certain time. The averaged response (recovery) time of the La-SnO_2_ NB device and its counterpart were measured at concentrations of 70, 100, 200, and 300 ppm and the results are listed in Table 1.

**Table 1:** The performance of two NB sensors towards different concentrations of VOC gases.

Concentrations		70 ppm		100 ppm		200 ppm		300 ppm			
Time		T_res_ (s)	T_rec_ (s)	T_res_ (s)	T_rec_ (s)	T_res_ (s)	T_rec_ (s)	T_res_ (s)	T_rec_ (s)	Average T_res_(s)	Average T_rec_(s)
Ethanediol	doped	9	7	12	4	15	5	16	6	13	5.5
	pure	4	9	5	3	3	17	10	9	5.5	9.5
Ethanol	doped	6	6	5	10	3	6	8	10	5.5	8.0
	pure	5	8	8	4	10	15	3	12	6.5	9.75
Acetone	doped	5	6	5	5	3	7	3	10	4.0	7.0
	pure	5	6	6	6	7	5	4	6	5.5	5.75

The La-SnO_2_ NB and pure SnO_2_ NB have a response (recovery) time of 13 s (5.5 s) and 5.5 s (9.5 s) to ethanediol, 5.5 s (8.0 s) and 6.5 s (9.75 s) to ethanol, 4.0 s (7.0 s) and 5.5 s (5.75 s) to acetone respectively at 230 °C. For ethanediol, the response time of La-SnO_2_ NB sensor is slightly larger than that of the pure SnO_2_ one, which is related to the fact that for the reasons discussed in Section 3.3, the La-SnO_2_ NB sensor shows a higher response to ethanediol. However, the response (recovery) times of the two sensors to ethanol and acetone are similar. The response time and recovery time of nanoscale devices is obviously smaller than that of traditional films [22,23] so nanoscale devices are suitable as a core part of a gas sensor.

### 3.3. Gas Sensing Mechanism

The La-SnO_2_ gas sensor is a surface resistance control type. In the crystal structure, Sn and O often deviate from the stoichiometric ratio, resulting in the formation of a donor level in which its forbidden band is close to the conduction band. The donor electrons can excite to the conduction band easily and participate in conducting. Oxygen molecules always adsorb on the surface of gas sensor in clean air [24] because the affinity of oxygen is very strong. The oxygen molecules on the surface gain electrons and form an acceptor level so that the surface of the gas sensor is negatively charged. The process is as follows:

(1) O_2_ (gas) ⇔ O_2_ (adsorption)

(2) O_2_ (adsorption) + e^−^ ⇔ O_2_^−^ (adsorption)

(3) O_2_^−^ (adsorption) + e^−^ ⇔ 2O^−^ (adsorption)

(4) O_2_^−^ (adsorption) + e^−^ ⇔ O^2−^ (adsorption)

The chemisorbed oxygen ions (including O_2_^−^, O^−^ and O^2−^) react with C_2_H_6_O_2_ and then produce electrons:

(5) 5O_2_^−^ + 2C_2_H_6_O_2_ = 6H_2_O + 4CO_2_ +5e^−^

(6) 10O^−^ + 2C_2_H_6_O_2_ = 6H_2_O + 4CO_2_ +10e^−^

(7) 5O^2−^ + 2C_2_H_6_O_2_ = 6H_2_O + 4CO_2_ +10e^−^

As a result, the sensitivity and selectivity of the La-SnO_2_ NB device are enhanced. In addition, La^3+^ ions have an influence on SnO_2_ described as Equation (1) and then oxygen vacancies are created during the transformation of LaO^+^ to La_2_O_3_ on the SnO_2_ surface [25]. At the same time, La_2_O_3_, as the ultimate heat-treatment product, presents a strong surface basicity which leads to a number of peroxide O_2_^2−^ ions chemisorbed on the La_2_O_3_ surface. The chemisorbed peroxide O_2_^2−^ could dissociate to oxygen ions (O^−^) that may transfer to the surface oxygen vacancies of SnO_2_ [25]. On the other hand, it could trigger an H-abstraction chemical reaction which could lower the reaction energy of the oxidation of hydrated carbon. The chemical reactions during this phase can be explained by the following equations:

(8) La^3+^ + H_2_O(g) → LaO^+^ + H^+^

(9) O_O_^×^ ↔ V_O_^••^ + 2e^−^ + 1/2O_2_

(10) 2LaO^+^ + O_O_^×^ → La_2_O_3_ + V_O_^••^ + 2e^−^

Based on the Equations (8)–(10), the amount of the adsorbed O^−^ species decreases as part of the surface of SnO_2_ is covered by the La_2_O_3_ [26] and then the reaction become slower. As a result the response time toward ethanediol is longer than the recovery time. Perhaps La^3+^ ions have different influences on ethanediol, ethanol and acetone. Therefore, the response time to ethanol (acetone) is not an anomalous result.

## 4. Conclusions

In conclusion, La-SnO_2_ NBs and pure SnO_2_ NBs were synthesized in a tube furnace by thermal evaporation at 1350 °C with Ar carrier gas (30 sccm, 125 Torr), and high sensitive single SnO_2_ and La-SnO_2_ NB sensors were thus developed. It is found that the La-SnO_2_ NB device exhibits a higher sensitivity of 8.76 to100 ppm of ethanediol at 230 °C, which is the highest sensitivity among the three tested VOC gases. The higher response is related to the selective catalysis of doped La^3+^ ions. This route can be extended to other metallic oxides semiconductors to promote their response, sensitivity, and selectivity towards some special toxic, inflammable or hazardous gases and VOC liquids.
